# Long-Term Oncologic Outcomes of Induction Chemotherapy Followed by Surgery Versus Upfront Surgery in Oral Cavity Squamous Cell Carcinoma

**DOI:** 10.3390/cancers18101590

**Published:** 2026-05-14

**Authors:** Yu-Fu Su, Po-Chien Shen, Yi-Jan Hsia, Wen-Yen Huang, Jing-Min Hwang

**Affiliations:** 1 Department of Radiation Oncology, Tri-Service General Hospital, National Defense Medical University, Taipei 114, Taiwan; yufusu1122@gmail.com (Y.-F.S.); tedsen2002.be10@nycu.edu.tw (P.-C.S.); hwyyi@yahoo.com.tw (W.-Y.H.); 2 Medical Department and Division of Oral Maxillofacial Surgery, Taipei Tzuchi Hospital, The Buddhist Tzuchi Medical Foundation, New Taipei City 231, Taiwan; yjhsia@yahoo.com.tw; 3 Department of Radiation Oncology, Taipei Tzuchi Hospital, The Buddhist Tzuchi Medical Foundation, New Taipei City 231, Taiwan

**Keywords:** oral cavity squamous cell carcinoma, induction chemotherapy, surgery, prognostic factors, surgical margins

## Abstract

Background: The optimal role of induction chemotherapy (IC) in the management of oral cavity squamous cell carcinoma (OCSCC) remains controversial. This study compared oncologic outcomes between IC followed by surgery and concurrent chemoradiotherapy (CCRT) and upfront surgery followed by adjuvant CCRT. Methods: We retrospectively analyzed 98 patients with OCSCC treated between 2011 and 2017. Overall survival (OS), cancer-specific survival (CSS), and local control (LC) were evaluated using Kaplan–Meier survival analysis and Cox proportional hazards models to identify prognostic factors. Results: Fifty patients received IC and 48 underwent upfront surgery. With a median follow-up of 77.8 months, no significant differences in OS, CSS, or LC were observed between treatment groups (OS: HR 1.31, *p* = 0.415; CSS: HR 1.36, *p* = 0.421; LC: HR 1.29, *p* = 0.475). Positive surgical margins independently predicted inferior OS, CSS, and LC, while extracapsular spread was independently associated with inferior CSS. Although tumor downstaging was frequently observed after IC, it did not translate into survival benefit. Conclusions: IC followed by surgery was associated with no statistically significant differences in oncologic outcomes compared with upfront surgery followed by adjuvant CCRT. Prognosis was primarily determined by pathological risk factors rather than treatment sequence.

## Simple Summary

The optimal treatment sequence for advanced oral cavity squamous cell carcinoma remains controversial. Some clinicians use induction chemotherapy before surgery to shrink tumors and potentially preserve function, while others prefer immediate surgery followed by postoperative therapy. In this study of 98 patients treated at a single institution, we found that survival outcomes were similar between patients receiving induction chemotherapy followed by surgery and those undergoing upfront surgery. Importantly, prognosis was mainly determined by pathological factors such as surgical margin status rather than treatment sequence. These findings suggest that treatment decisions should focus on achieving complete tumor removal and careful patient selection.

## 1. Introduction

Oral cavity squamous cell carcinoma (OCSCC) represents a major subtype of head and neck malignancies worldwide, with particularly high incidence rates reported in Asian populations due to exposure to established carcinogenic factors such as tobacco use, alcohol consumption, and betel nut chewing [1,2]. In Taiwan, oral cancer ranks as the fourth most common malignancy among men, contributing significantly to cancer-related morbidity and mortality [3]. The burden of this disease is intensified by its tendency to present at an advanced stage and the complex anatomical and functional roles of the oral cavity.

Primary surgical resection is the current standard of care for resectable advanced-stage OCSCC, followed by risk-adapted postoperative radiotherapy or concurrent chemoradiotherapy (CCRT) based on pathological risk factors. This approach provides high rates of locoregional control and disease-specific survival, especially when negative margins are achieved [4,5,6]. However, major surgical procedures may lead to substantial functional and cosmetic consequences, such as impaired speech, swallowing difficulties, and facial deformity, all of which can adversely affect long-term quality of life [7,8,9].

Induction chemotherapy (IC) has been proposed as a neoadjuvant strategy aimed at tumor downstaging, facilitating more conservative surgery, and potentially improving organ preservation and cosmesis to address these challenges [10]. In addition, IC has been proposed as a strategy to evaluate initial tumor responsiveness and to guide the selection of patients who may be appropriate for definitive non-surgical CCRT [11,12]. Although IC can induce tumor shrinkage and improve operability in some patients with oral cancer, its effects on surgical margins and overall survival (OS) remain inconclusive [13,14]. However, the survival advantage of IC over upfront surgery remains controversial, as other studies have shown no significant improvement in OS or locoregional control [11,15].

An earlier matched cohort study compared surgical management with organ-preservation approaches incorporating IC and CCRT in patients with oral cavity squamous cell carcinoma [16]. As that study addressed a different clinical question, direct comparison with the present study should be interpreted cautiously. In addition to oncologic outcomes, studies have shown that the extent of surgical resection and reconstruction techniques are closely associated with patient-reported quality of life, particularly in speech, swallowing, and appearance domains [17]. Collectively, these findings highlight the ongoing debate regarding the optimal role, timing, and patient selection of IC in the management of advanced OCSCC.

However, previous studies evaluating induction chemotherapy in OCSCC have been limited by heterogeneous treatment strategies, variations in patient selection, and relatively short follow-up durations. In addition, real-world comparative data regarding long-term oncologic outcomes between induction chemotherapy-based approaches and upfront surgery remain limited. Therefore, further investigation is warranted to clarify the clinical role of induction chemotherapy in contemporary OCSCC management.

Given these disparities, real-world data are essential to evaluate treatment strategies outside the constraints of controlled clinical trials. In our institution, a substantial proportion of patients with OCSCC received either IC followed by surgery and CCRT or upfront surgery followed by adjuvant CCRT. This study aimed to compare the oncologic outcomes between these two approaches and identify clinicopathologic factors that influence prognosis. It was hypothesized that the induction-first strategy could achieve acceptable disease control compared with the surgery-first approach while potentially reducing surgical morbidity and improving postoperative quality of life.

## 2. Materials and Methods

### 2.1. Study Population

A total of 467 patients with oral cavity cancer were initially identified from the institutional database during the study period. Patients were screened according to predefined eligibility criteria. Inclusion criteria included curative-intent treatment, M0 disease status, newly diagnosed primary oral cavity cancer, a total radiotherapy dose > 5000 cGy, Eastern Cooperative Oncology Group (ECOG) performance status of 0–2, completion of the planned treatment modality, and adequate follow-up duration.

Patients were excluded if they received surgery alone (*n* = 170), surgery with systemic therapy only (*n* = 105), radiotherapy with systemic therapy only (*n* = 20), other treatment modalities, or had incomplete data or treatment (*n* = 74). After applying these criteria, 98 eligible patients were included in the final analysis.

This retrospective study reviewed the medical records of 98 patients with OCSCC who received curative-intent treatment at our institution between January 2011 and December 2017. Patients were categorized into two groups based on their initial treatment strategy: those who received IC followed by surgery and CCRT and those who underwent upfront surgery followed by CCRT.

Baseline demographic and clinical characteristics—including age, sex, tumor site, surgical margin status, and presence of extracapsular spread (ECS)—were collected for comparative analysis.

### 2.2. Treatment Protocol

Treatment strategies were determined through multidisciplinary team (MDT) discussions involving head and neck surgeons, oral and maxillofacial surgeons, radiation oncologists, medical oncologists, radiologists, and pathologists. Decisions regarding induction chemotherapy, surgical management, and postoperative adjuvant therapy were individualized based on tumor extent, resectability, patient condition, and anticipated functional and cosmetic outcomes.

Induction chemotherapy primarily consisted of the TPF regimen, including docetaxel (40 mg/m^2^), cisplatin (40 mg/m^2^), and 5-fluorouracil (750–1000 mg/m^2^), administered every three weeks for three cycles. In selected patients, cetuximab was administered with an initial loading dose of 400 mg/m^2^ followed by 250 mg/m^2^ weekly. For patients who were considered unsuitable for cytotoxic chemotherapy due to advanced age or comorbidities, cetuximab was used as an alternative systemic therapy.

Concurrent systemic therapy during radiotherapy consisted of weekly cisplatin (30–40 mg/m^2^) or carboplatin, with cetuximab used as an alternative in patients unable to tolerate platinum-based chemotherapy.

Postoperative radiotherapy or concurrent chemoradiotherapy was delivered according to risk stratification. High-risk patients (e.g., positive surgical margin or extracapsular spread) received 6600 cGy in 33 fractions over 6–7 weeks. Patients with intermediate-risk features received 6000 cGy in 30 fractions, while low- to intermediate-risk areas received 5000 cGy in 25 fractions.

Radiotherapy was delivered using a linear accelerator with image-guided radiotherapy. Treatment planning was performed using a computerized treatment planning system. Intensity-modulated radiotherapy (IMRT) with a sequential boost technique and routine image verification using cone beam computed tomography (CBCT) was applied in all patients.

### 2.3. Outcome Measures

The primary outcomes of interest in this study included OS, cancer-specific survival (CSS), and local control (LC).

OS was defined as the duration from the date of diagnosis to death from any cause or to the date of the last follow-up for patients who were still alive.

CSS was defined as the time interval from diagnosis to death caused by the index cancer, while deaths unrelated to the disease were treated as censored events at the time of occurrence.

LC was defined as the interval from diagnosis to the first documented evidence of local or regional tumor recurrence, confirmed by imaging studies, histopathological findings, or clinical evaluation.

### 2.4. Statistical Analysis

Baseline patient characteristics were summarized using descriptive statistical methods. Survival outcomes were analyzed using the Kaplan–Meier method, and differences between groups were evaluated using the log-rank test. Potential prognostic variables were examined using Cox proportional hazards regression models. Variables showing potential associations in the univariable analysis (*p* < 0.10), together with clinically relevant variables, were considered for inclusion in the multivariable analysis. The final multivariable models included treatment modality, tumor stage, surgical margin status, and extracapsular spread. These analyses were conducted to explore possible associations with clinical outcomes and to provide additional context for interpretation of treatment results. A two-sided *p*-value < 0.05 was considered statistically significant. This retrospective study was approved by the Institutional Review Board of Taipei Tzu Chi Hospital (IRB No. 13-IRB108) on 7 October 2024. The requirement for informed consent was waived due to the retrospective design of the study and the use of anonymized patient data.

## 3. Results

### 3.1. Patient Characteristics

A total of 98 patients were included, with 50 (51%) in the IC group and 48 (49%) in the surgery-first group as shown in Table 1. The median age at diagnosis was 55 years (range, 28–81 years). The buccal mucosa was the most common primary tumor site (39.7%), followed by the oral tongue (28.5%), gingiva (16.3%), hard palate (6.1%), lip (6.1%), and floor of the mouth (3%).

Histologically, most tumors were conventional squamous cell carcinomas (96%). The rare variants included verrucous carcinoma (2%), clear cell SCC (1%), and adenoid cystic carcinoma (1%).

Patients in the induction chemotherapy group had a higher proportion of advanced disease, including T4b tumors and stage IVB disease, compared with the upfront surgery group.

### 3.2. Treatment Modalities

Patients in the IC group received a median of three cycles of induction therapy, and most patients completed the planned course as scheduled. Concurrent systemic therapy during radiotherapy mainly consisted of weekly cisplatin or carboplatin. Only one patient received up to eight cycles of cetuximab during radiotherapy. Overall, treatment tolerance was acceptable, and all patients completed their planned course of radiotherapy and systemic therapy without significant interruptions. The median adjuvant radiotherapy dose was 64 Gy (range, 50–70 Gy).

### 3.3. Follow-Up and Outcomes

**Figure 1:**
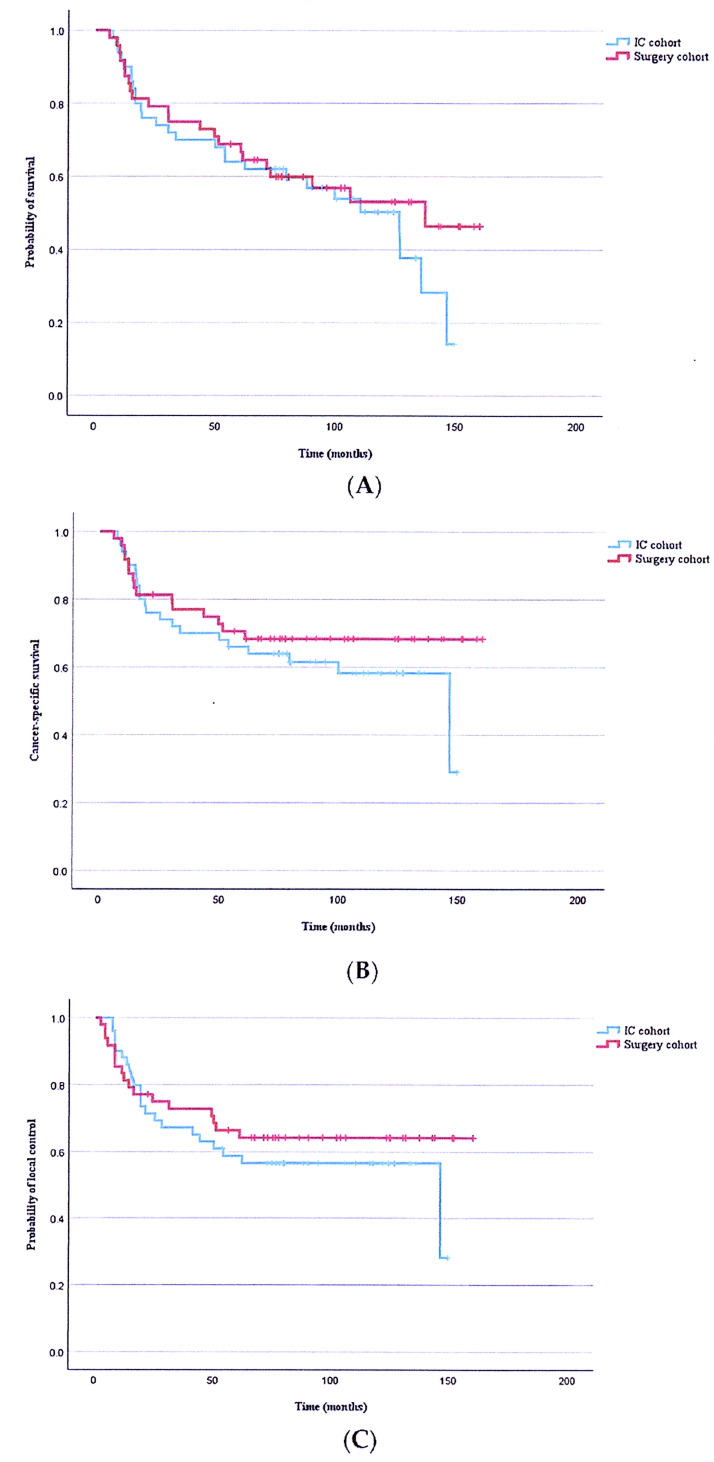
Kaplan–Meier survival curves of the IC and surgery cohorts. (**A**) Overall survival (*p* = 0.416). (**B**) Cancer-specific survival (*p* = 0.339). (**C**) Local control (*p* = 0.419). No statistically significant differences were observed between the two treatment groups across all endpoints.

With a median follow-up of 77.8 months (range, 6–160 months), no significant differences in OS, CSS, or LC were observed between the two treatment groups (Figure 1). Tumor downstaging was frequently observed in the IC group. However, the present study did not directly evaluate its association with surgical extent or postoperative functional outcomes. The LC rates between the two groups were comparable.

### 3.4. Prognostic Factor

Univariable analysis revealed that surgical margin status was significantly associated with OS (HR, 3.09; 95% CI, 1.29–7.39; *p* = 0.011), CSS (HR, 3.67; 95% CI, 1.51–8.93; *p* = 0.004), and LC (HR, 2.88; 95% CI, 1.19–6.94; *p* = 0.019) (Table 2). In contrast, age, sex, tumor stage, treatment modality, and ECS were not significantly associated with survival outcomes (all *p* > 0.05).

In the multivariable Cox regression model (Table 3), positive surgical margin remained an independent adverse prognostic factor for OS (HR, 4.12; 95% CI, 1.62–10.51; *p* = 0.003), CSS (HR, 6.25; 95% CI, 2.37–16.47; *p* < 0.001), and LC (HR, 3.98; 95% CI, 1.57–10.11; *p* = 0.004). Notably, ECS also emerged as an independent predictor of inferior CSS (HR, 2.39; 95% CI, 1.12–5.12; *p* = 0.025), whereas OS or LC did not reach significance. Other factors, such as treatment modality and stage, were not independently associated with survival outcomes.

### 3.5. Pathologic Findings After IC

Among patients who received induction chemotherapy followed by surgery (*n* = 50), additional analysis was performed to evaluate changes between clinical and postoperative pathologic staging.

Downstaging of the primary tumor (defined as clinical T stage greater than pathologic T stage) was observed in 27 of 50 patients (54.0%). Downstaging of nodal status was observed in 23 of 50 patients (46.0%). Overall, either T or N downstaging occurred in 37 of 50 patients (74.0%), and simultaneous downstaging of both T and N stages was observed in 13 patients (26.0%). These findings indicate that induction chemotherapy was associated with measurable tumor response in a substantial proportion of patients.

## 4. Discussion

In our study, no statistically significant differences in OS, CSS, or LC were observed between IC followed by surgery and upfront surgery with adjuvant CCRT. However, these findings should be interpreted cautiously given the limited sample size and potential for insufficient statistical power to detect modest differences. Although tumor downstaging was frequently observed after induction therapy, it did not improve survival or local control.

Prognosis was primarily driven by pathologic risk rather than treatment sequence. Positive surgical margins independently predicted worse OS, CSS, and LC, consistent with contemporary reports that identified margin involvement as a dominant determinant of OCSCC outcome [18,19,20,21]. ECS was also independently associated with inferior CSS, aligning with systematic reviews and clinical series that link ECS to aggressive tumor biology and higher risks of locoregional recurrence and distant metastasis [22,23,24,25,26]. Recent molecular and tumor microenvironment studies have further highlighted the biological heterogeneity of OCSCC and its association with tumor progression, immune suppression, and prognosis [27,28]. These findings reinforce the importance of achieving negative margins during initial resection and support the selective use of IC to facilitate complete excision in cases requiring tumor reduction, with postoperative treatment intensification considered for patients with margin involvement or ECS. It should be noted that the prognostic factor analyses in this study were exploratory in nature and were intended to provide supportive clinical context rather than to establish definitive predictors of outcome, given the relatively small sample size and the limited statistical precision reflected by the wide confidence intervals.

Beyond oncologic outcomes, functional and aesthetic preservation remains a critical aspect of OCSCC management, particularly for tumors involving the lower face, lips, or tongue. Extensive composite resections, such as mandibulectomy or glossectomy, often result in significant speech, swallowing, and masticatory impairments, as well as cosmetic disfigurement. Advances in reconstructive surgery, particularly the use of free flap reconstruction techniques, have substantially improved postoperative function and quality of life. Patients undergoing free flap reconstruction achieved better long-term oral intake, speech intelligibility, swallowing, and aesthetic outcomes than those treated with locoregional flaps or primary closure [29,30].

Previous studies have suggested that induction chemotherapy may facilitate tumor downstaging, potentially allowing less extensive surgery and greater opportunities for organ preservation in selected patients [31]. However, the present study did not directly evaluate surgical extent, reconstructive complexity, or functional outcomes; therefore, the clinical significance of tumor downstaging should be interpreted cautiously. In the present study, additional analysis of pathologic staging changes demonstrated measurable tumor response following induction chemotherapy. Downstaging of either the primary tumor or nodal disease was observed in 74% of patients, with simultaneous downstaging of both components in 26% of cases. These findings suggest the biological activity of induction chemotherapy and are consistent with prior reports demonstrating tumor response after neoadjuvant therapy in oral cavity cancer. In a phase II randomized controlled trial, IC facilitated mandibular preservation in patients with operable OCSCC without compromising oncologic outcomes [31]. Other contemporary studies also indicate that IC or neoadjuvant strategies can enable resection in previously unresectable tumors and may improve functional recovery among responders, although consistent survival benefits remain unproven [32,33,34]. Collectively, these findings suggest that IC may serve as an adjunct to facilitate resectability in selected patients, while meticulous surgical resection with free flap reconstruction remains the cornerstone of curative treatment.

Several limitations of the present study should be acknowledged. Tumor subsite has been recognized as an important prognostic factor in OCSCC, although findings have been inconsistent across studies [35,36,37]. Tongue cancer has been associated with higher rates of adverse pathological features, including perineural invasion and lymphovascular invasion, compared with other oral cavity subsites [35,38,39]. However, some studies have reported comparable or even superior overall survival for tongue cancer after adjusting for stage and treatment factors, while others have identified hard palate and alveolar ridge as the subsites with the worst prognosis [36,37,40]. These discrepancies may reflect differences in study populations, analytical methods, and the specific endpoints examined. However, detailed subgroup analysis according to tumor subsite was not feasible because tumor location was incompletely documented in the original medical records. In addition, several sources of potential bias inherent to retrospective real-world analyses should be acknowledged. Treatment allocation in this study was determined through multidisciplinary team discussions based on tumor characteristics, patient condition, and anticipated functional outcomes, which may introduce selection bias associated with non-randomized clinical decision-making. Similarly, the use of cetuximab in patients considered unsuitable for cytotoxic chemotherapy due to advanced age or comorbidities reflects treatment selection based on clinical fitness and may introduce confounding by indication. Furthermore, variability in the administration of concurrent systemic therapy and postoperative treatment intensity reflects the heterogeneity of routine clinical practice rather than standardized protocol-driven management. Outcome definitions may also introduce uncertainty. Overall survival was calculated from the date of diagnosis, and local control was defined based on the time of documented recurrence, both of which may be influenced by variations in treatment intervals and follow-up schedules in a retrospective setting. These methodological limitations are common in observational studies and underscore that the findings of the present analysis should be interpreted cautiously within the context of real-world clinical data.

## 5. Conclusions

Induction chemotherapy followed by surgery was not associated with statistically significant differences in oncologic outcomes compared with upfront surgery followed by adjuvant concurrent chemoradiotherapy in patients with OCSCC. Prognosis was primarily determined by pathological risk factors, particularly surgical margin status and extracapsular spread, rather than treatment sequence. These findings support individualized treatment selection and highlight the importance of achieving negative surgical margins.

## Figures and Tables

**Table 1:** Baseline characteristics of the study population by treatment modality.

Characteristic	Induction (*n* = 50)	Surgery (*n* = 48)	Total (*n* = 98)
**Age, median (range)**	54.5 (28–81)	55.5 (38–77)	55.0 (28–81)
**Sex**			
Male	48 (96.0%)	44 (91.7%)	92 (93.9%)
Female	2 (4.0%)	4 (8.3%)	6 (6.1%)
**Clinical T-stage**			
T1	2	6	8
T2	4	17	21
T3	2	5	7
T4a	26	17	43
T4b	16	3	19
**Clinical N-stage**			
N0	11	22	33
N1	17	14	31
N2a	0	1	1
N2b	17	9	26
N2c	4	2	6
N3	1	0	1
**AJCC Clinical Stage (7th ed.)**			
I	1	6	7
II	1	8	9
III	2	8	10
IVA	30	23	53
IVB	16	3	19

Data are presented as number (%) unless otherwise indicated. Clinical staging was determined according to the 7th edition of the American Joint Committee on Cancer (AJCC) staging system. OCSCC, oral cavity squamous cell carcinoma. Category headings indicate subgroup titles and do not contain data.

**Table 2:** Univariable Cox proportional hazards analysis of clinical factors associated with survival outcomes.

	Overall Survival HR (95% CI), *p* Value	Cancer-Specific Survival HR (95% CI), *p* Value	Local Control HR (95% CI), *p* Value
Treatment: induction vs. surgery	1.27 (0.72–2.23), 0.417	1.38 (0.71–2.68), 0.342	1.30 (0.69–2.44), 0.424
Age >55 vs. ≤55	1.42 (0.81–2.50), 0.223	1.29 (0.67–2.48), 0. 450	1.17 (0.62–2.19), 0.634
Gender: male vs. female	4.02 (0.55–29.14), 0.169	2.79 (0.38–20.38), 0.312	1.40 (0.34–5.82), 0.644
Stage: late (stage IV) vs. early	1.14 (0.61–2.14), 0.685	1.73 (0.75–4.00), 0.2 00	1.34 (0.63–2.85), 0.445
Surgical Margin: positive vs. negative	3.09 (1.29–7.39), **0.011**	3.67 (1.51–8.93), **0.004**	2.88 (1.19–6.94), **0.019**
ECS: positive vs. negative	1.47 (0.75–2.88), 0.261	1.87 (0.90–3.87), 0.095	1.66 (0.81–3.41), 0.168

Univariable Cox proportional hazards analysis was performed to evaluate associations between clinical factors and overall survival (OS), cancer-specific survival (CSS), and local control (LC). The results are expressed as hazard ratios with 95% confidence intervals. Statistically significant associations (*p* < 0.05) are indicated in bold.

**Table 3:** Multivariable Cox regression analysis of factors associated with survival outcomes.

	Overall Survival HR (95% CI), *p* Value	Cancer-Specific Survival HR (95% CI), *p* Value	Local Control HR (95% CI), *p* Value
Treatment: induction vs. surgery	1.31 (0.69–2.50), 0.415	1.36 (0.65–2.84), 0.421	1.29 (0.64–2.62), 0.475
Stage: late (stage IV) vs. early stage	1.18 (0.58–2.41), 0.641	1.96 (0.78–4.96), 0.154	1.44 (0.63–3.31), 0.391
Surgical margin: positive vs. negative	4.12 (1.62–10.51), **0.003**	6.25 (2.37–16.47), **< 0.001**	3.98 (1.57–10.11), **0.004**
ECS: positive vs. negative	1.78 (0.89–3.57), 0.105	2.39 (1.12–5.12), **0.025**	2.01 (0.96–4.22), 0.064

Multivariable Cox regression models were applied to evaluate factors independently associated with OS, CSS, and LC. Variables with *p* < 0.10 in univariable analysis and clinically relevant variables were included in the multivariable models. Effect estimates are presented as hazard ratios (HRs) with corresponding 95% confidence intervals (CIs). Statistically significant results (*p* < 0.05) are highlighted in bold.

## Data Availability

The data presented in this study are available from the corresponding author upon reasonable request.
